# Marinopyrrole Derivatives as Potential Antibiotic Agents against Methicillin-Resistant *Staphylococcus aureus* (III)

**DOI:** 10.3390/md12052458

**Published:** 2014-04-30

**Authors:** Yan Liu, Nina M. Haste, Wdee Thienphrapa, Jerry Li, Victor Nizet, Mary Hensler, Rongshi Li

**Affiliations:** 1 Department of Pharmaceutical Sciences, Center for Drug Discovery, College of Pharmacy, University of Nebraska Medical Center, 986805 Nebraska Medical Center, Omaha, NE 68198, USA; E-Mail: Yan.Liu@unmc.edu; 2 Cancer Genes and Molecular Regulation Program, Buffett Cancer Center, University of Nebraska Medical Center, 986805 Nebraska Medical Center, Omaha, NE 68198, USA; 3 Department of Drug Discovery, Chemical Biology & Molecular Medicine Program, H. Lee Moffitt Cancer Center and Research Institute, 12902 Magnolia Drive, Tampa, FL 33612, USA; E-Mail: Jerry.Li@ucsf.edu; 4 Skaggs School of Pharmacy and Pharmaceutical Sciences, University of California San Diego, La Jolla, CA 92093, USA; E-Mails: nhaste@ucsd.edu (N.M.H.); vnizet@ucsd.edu (V.N.); 5 Department of Pediatrics, University of California San Diego, La Jolla, CA 92093, USA; E-Mails: wdee.ucsd@gmail.com (W.T.); mhensler@ucsd.edu (M.H.); 6 Department of Oncologic Sciences, Morsani College of Medicine, University of South Florida, 12901 Bruce B. Downs, Tampa, FL 33612, USA

**Keywords:** antibiotics, non-symmetrical marinopyrroles, MRSA, SAR

## Abstract

The marine natural product, marinopyrrole A (**1**), was previously shown to have significant antibiotic activity against Gram-positive pathogens, including methicillin-resistant *Staphylococcus aureus* (MRSA). Although compound (**1**) exhibits a significant reduction in MRSA activity in the presence of human serum, we have identified key modifications that partially restore activity. We previously reported our discovery of a chloro-derivative of marinopyrrole A (**1a**) featuring a 2–4 fold improved minimum inhibitory concentration (MIC) against MRSA, significantly less susceptibility to serum inhibition and rapid and concentration-dependent killing of MRSA. Here, we report a novel fluoro-derivative of marinopyrrole A (**1e**) showing an improved profile of potency, less susceptibility to serum inhibition, as well as rapid and concentration-dependent killing of MRSA.

## 1. Introduction

Since we reported the synthesis of novel non-symmetrical marinopyrrole derivatives retaining their potent activity against methicillin-resistant *Staphylococcus aureus* (MRSA), yet less susceptible to human serum inhibition [1], several research publications on the topic of marinopyrroles have appeared [2,3,4,5,6,7]. Biosynthesis of marinopyrrole A via an *N*,*C*-bipyrrole homocoupling catalyzed by two flavin-dependent halogenases was reported by the Moore group [2], and racemic marinopyrrole B by total synthesis and a review of the marinopyrroles were reported by the Clive group [3,4]. After optimization of the key step to avoid the formation of an oxazepine byproduct [5] that was reported in our first total synthesis of marinopyrroles [8], we published the most potent symmetrical marinopyrrole derivative against MRSA and methicillin-resistant *Staphylococcus epidermidis* (MRSE) [6]. Recently we reported a series of cyclic marinopyrroles as disruptors of Mcl-1 and Bcl-x_L_ binding to Bim [7] and a series of novel marinopyrroles with potential as anticancer agents [9].

The World Health Organization recognizes antibiotic resistance as a serious threat to human health [10]. The global crisis of antibiotic resistance has spread rapidly over the last several decades, with multidrug-resistant MRSA as a major cause of serious infections in the United States [11,12,13,14,15]. From 1999 to 2005, estimated MRSA hospitalizations in the U.S. more than doubled, increasing from 127,000 to 280,000, and accounted for roughly 94,000 infections and close to 19,000 deaths in 2005 [16]. That same year, more people in the U.S. died from MRSA infections than HIV/AIDS (16,000 people). MRSA infections cost U.S. hospitals between $3.2 and $4.2 billion annually [17]. Recent survey documents have shown that MRSA remains one of the most prevalent multidrug-resistant organisms causing healthcare-associated infections, and the MRSA prevalence in 2010 is higher than that reported in 2006 [18]. The introduction of new MRSA antibiotics to clinical practice has been limited primarily to the oxazolidinone, linezolid [19], in 2000, the lipopeptide, daptomycin [20], in 2003, and ceftaroline [21]. Vancomycin remains the most commonly used first line treatment against MRSA. However, overreliance on this drug has resulted in an increase in MRSA with reduced susceptibility to vancomycin [22,23]. The minimum inhibitory concentration (MIC) shift (“the MIC creep”) for vancomycin has been especially noticeable in MRSA compared to other *S. aureus* [24]. In fact, vancomycin efficacy continues to decline, due to pathogen-developed resistance [22,24]. Instances of daptomycin [25] and linezolid [26,27] resistance have also surfaced. There are now several late-stage products in development, including tedizolid, dalbavancin, oritavancin and ceptobiprole. Although these antibiotics may add to the arsenal for combating MRSA resistance, bacteria inevitably develop resistance to all antibacterial agents that are introduced to the clinic [28]. Novel antibiotic agents of new structural classes and further advances in discovery research are urgently needed to overcome the problem of MRSA resistance.

The relative abandonment of the discovery and development of antibiotics by the pharmaceutical industry has opened opportunities for academic researchers to discover new antibiotics that treat these increasingly problematic infections. Here, we report our design and synthesis of novel marinopyrrole derivatives with excellent antibiotic activity against MRSA, but only limited serum inactivation.

## 2. Results and Discussion

### 2.1. Synthesis and Structural Activity Relationships of Non-Symmetrical Marinopyrrole Derivatives

**Chart 1:**
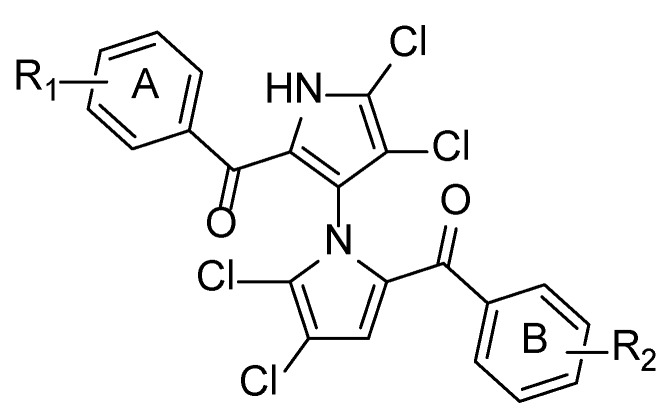
Marinopyrroles.

We classified marinopyrroles as “symmetrical” and “asymmetrical/non-symmetrical” in our previous publication to facilitate structure-activity relationship (SAR) discussions [1]. “Symmetrical” derivatives bear the same substituents and patterns on both phenyl Rings A and B attached to the carbonyl groups, while “non-symmetrical” marinopyrroles are those with different substituents for Rings A and B (Chart 1). As we envisaged that the “non-symmetrical” marinopyrrole derivatives should have different and possibly more favorable biological activity than their symmetrical counterparts, in particular, the molecules with diverse functional groups decorated on this unique 1,3-bispyrrole system may adopt specific conformations, due to the restricted free rotation of the chiral axis. Indeed, a series of novel non-symmetrical marinopyrrole derivatives that we designed and synthesized showed potent anti-MRSA activity with a superior antibiotic profile to the parent marinopyrrole A (**1**) [1].

**Scheme 1:**
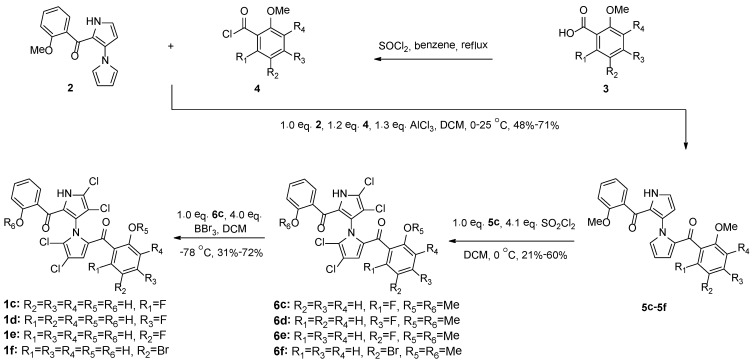
Synthesis of novel non-symmetrical marinopyrrole derivatives.

**Figure 1:**
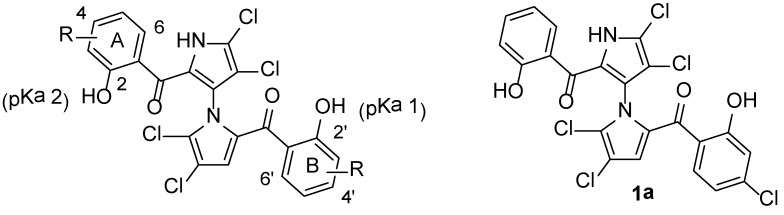
Physicochemical properties and structure-activity relationship (SAR) of marinopyrrole derivatives against methicillin-resistant *Staphylococcus aureus* (MRSA).

**Figure 2:**
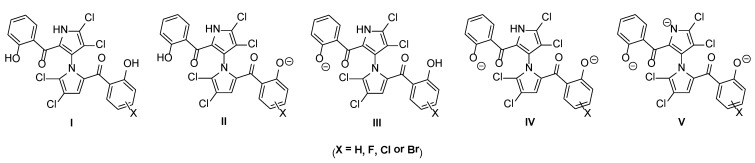
pH-dependent microspecies of marinopyrroles.

To continue our efforts of structure-activity relationship (SAR) optimization, we designed and synthesized a series of novel non-symmetrical marinopyrroles and evaluated their anti-MRSA activity. As shown in and Scheme 1, while Ring A was kept constant, substitutions with different halogen (F, Cl and Br) in the different position of Ring B were examined. Indeed, the effects of different halogen (F, Cl and Br) in Ring B on SARs, physicochemical properties and pH-dependent microspecies are observed, as detailed in Figure 1 and Figure 2.

Chemistries to access both symmetrical and non-symmetrical marinopyrrole derivatives have been reported [1,6,8,29]. Briefly, Friedel–Crafts arylation of mono acylated bispyrrole **2** [1,29] with the acid chlorides, **4**, generated *in situ* from the corresponding carboxylic acids, **3**, with thionyl chloride, afforded a series of marinopyrrole precursors, **5c**–**5f**, in a 48%–71% yield. A novel series of non-symmetrical marinopyrrole derivatives, **6c**–**6f**, were obtained in a 21%–60% yield by tetrachlorination of the corresponding **5c**–**5f** using 4.1 equivalents of sulfuryl chloride (SO_2_Cl_2_) in DCM at 0 °C. Demethylation of **6c**–**6f** using BBr_3_ in DCM at −78 °C furnished **1c**–**1f** in a 31%–72% yield (Scheme 1).

**Table:** 

Compound	MW ^f^	p*K*_a_ 1 *^a^*	p*K*_a_ 2 *^a^*	Clog *p ^a^*	THB *^c^*	THB + 20% Serum *^c^*
**1 (parent)**	510.15	7.8 *^b^*	8.4 *^b^*	5.6 *^b^*	0.75 *^d^*	94–188 *^d^*
**1a *^d^***	544.60	7.3	8.2	6.1	0.19–0.39 *^d^*	12.5–25 *^d^*
**1c**	528.14	7.2	8.2	5.7	3.13	ND *^e^*
**1d**	528.14	7.0	8.1	5.7	0.78	ND
**1e**	528.14	7.6	8.3	5.7	0.19–0.78	25–50
**1f**	589.05	7.5	8.2	6.4	1.56	ND
**Vanco** **^g^**	1485.72				0.85–1.7	0.85–1.7

*^a^* Calculated using ChemAxon Software Version 5.12.3 (ChemAxon, Budapest, Hungary); *^b^* reported in our previous paper [6]; *^c ^* MIC, minimum inhibitory concentration in μM in Todd-Hewitt broth (THB) in the absence or presence of 20% human serum; all data were generated from experiments repeated four times; *^d ^* results, except for the calculated p*K*_a_ and log *p*-values, from our previous paper [1] for SAR discussions; *^e^* not determined; ^f^ MW, molecular weight; ^g^ Vanco, vancomycin.

**Table:** 

Microspecies	1	1a	1c	1d	1e	1f
**I**	*85.3*/68.5/29.9 ^a^	*63.2*/38.4/9.7	*61.2*/36.3/9.0	*50.3*/26.7/5.9	*79.8*/59.4/21.3	*75.3*/52.7/16.7
**II**	*7.1*/14.3/24.8	*29.2*/44.4/45.0	*31.2*/46.5/45.7	*42.0*/56.1/48.8	*6.6*/12.4/17.6	*17.1*/30.0/38.0
**III**	*7.1*/14.3/24.8	*5.2*/8.0/8.1	*5.1*/7.6/7.4	*4.2*/5.6/4.8	*12.5*/23.4/33.4	*6.2*/11.0/13.9
**IV**	*0.6*/3.0/20.5	*2.4*/8.0/37.2	*2.6*/9.7/37.9	*3.5*/11.7/40.4	*1.0*/4.9/27.6	*1.4*/6.3/31.4
**V**	*0.0*/0.0/0.01	*0.0*/0.0/0.01	*0.0*/0.0/0.01	*0.0*/0.0/0.01	*0.0*/0.0/0.02	*0.0*/0.0/0.01

*^a^* Percentage (%) of microspecies distributions at pH 7.0/7.4/8.0, respectively, calculated using ChemAxon Software Version 5.12.3 (ChemAxon, Budapest, Hungary); numbers in italics are the percent of microspecies distributions at pH 7.0.

The anti-MRSA activity of the novel non-symmetrical marinopyrroles was evaluated against a USA300 strain of community-associated MRSA using marinopyrrole A (**1**) as a control. The MIC values of **1a** [(4-chloro-2-hydroxyphenyl)(4,4′,5,5′-tetrachloro-2′-(2-hydroxybenzoyl)-1′*H*-1,3′-bipyrrole-2-yl)methanone], which we reported previously [1], were used to facilitate the SAR discussions. Similar to the effects from chlorine substituted marinopyrroles [1], fluorine substitutions in Ring B have significant contributions to the antibacterial activity. Although non-symmetrical marinopyrrole with *ortho*-substitution of the carbonyl group (**1c**) is four times less potent than **1**, those marinopyrroles with *para*-(**1d**) and *meta*-fluoro (**1e**) substitutions display similar or better activities than **1**. One of the derivatives, (5-fluoro-2-hydroxyphenyl)(4,4′,5,5′-tetrachloro-2′-(2-hydroxybenzoyl)-1′*H*-1,3′-bipyrrol-2-yl)methanone (**1e**), exhibited potent antibacterial activity similar to that of **1a** [1]. The 1–4 fold improvement in antibacterial activity from the parent compound, **1**, was observed for this novel fluoro-substituted marinopyrrole derivative, **1e**, as shown in Figure 1. Compound **1f** with *meta*-bromo substitution also exhibited antibacterial activity, although the MIC value is two-fold less potent than **1**. Most significantly, not only did Compound **1e** show increased antibacterial activity compared to the parent compound, marinopyrrole A (**1**), but its activity was also less inhibited upon the addition of 20% human serum (MIC 25–50 μM *vs.* 94–188 μM). In comparison to contemporary MRSA agents, Compound **1e** is more potent than vancomycin against USA300 MRSA strain TCH1516 [30].

To understand the significant effects of the physicochemical properties on antibacterial activity, we calculated the p*K*_a_ 1, p*K*_a_ 2 and log *p* of all marinopyrrole derivatives (Figure 2). All fluoro-substituted marinopyrroles (**1c**–**1e**) have lower Clog *p*-values than **1**, while their chloro-(**1a**) or bromo-(**1f**) counterparts are up to half a log unit higher. Although p*K*_a_ 2 values do not vary much (8.1–8.4), the p*K*_a_ 1 values change from 7.0 to 7.8, due to the substitution of halogen atoms in different positions of Ring B. Careful analysis of p*K*_a_ data reveals that there are five microspecies, I–V, present, and their distributions depend on the pH, as shown in Figure 2. Although our MIC assays were performed at pH 7.0, microspecies distributions at pH 7.4 and 8.0 are also tabulated, as the latter conditions are usually used for other assays [7,9]. At pH 7.0, 50%–85% of all marinopyrroles are in the form of Microspecies I, with the parent marinopyrrole, **1**, being the most predominant (85%) Microspecies I (Figure 2); marinopyrroles **1** and **1e** have similar distributions of Microspecies II, 7.1% and 6.6%, respectively; the rest are increasing from 17% (**1f**) to 42% (**1d**) Microspecies II; the variation of Microspecies III distributions is small from 4.2% (**1d**) to 12.5% (**1e**); both Microspecies IV and V are from 0.0% to 3.5% at pH 7 and may be considered negligible. Microspecies I–IV distributions of marinopyrroles vary significantly at pH 7.4 and 8.0 (Figure 2). Microspecies I is found in the free hydroxyl form for both phenol groups, which can serve as both hydrogen bond donors and acceptors. Microspecies II and III can provide one free hydroxyl and one phenoxide group, as shown in Figure 2. The former can provide both a hydrogen bond donor and acceptor in Ring A and only a hydrogen bond acceptor or phenoxide for ionic interactions in Ring B, and *vice versa* for the latter. Microspecies IV has both phenol groups in phenoxide form, which are only available as hydrogen bond acceptors or for ionic interactions. The microspecies and their distributions at different pH should have a significant impact on their antibacterial activity.

**Figure 3:**
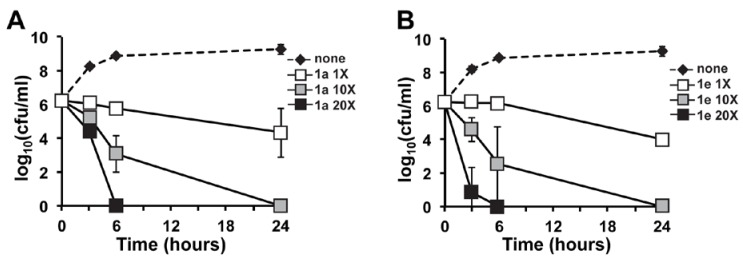
*In vitro* time-kill analysis for marinopyrrole derivatives **1a** (**A**) and **1e** (**B**) against the USA300 community-associated MRSA strain TCH1516. MRSA was subjected to increasing concentrations of 1× (0.39 μM), 10× (3.9 μM) and 20× (7.8 μM) the MIC of the assayed derivatives or the vehicle control (none). Both derivatives showed potent concentration-dependent killing kinetics. At 20× (7.8 μM), Derivative **1e** demonstrated more rapid bacterial killing at 3 h.

### 2.2. In Vitro Time-Kill of Marinopyrrole Derivative 1e

Our previous data showed that the marinopyrrole derivative, **1a**, exhibited rapid killing kinetics, and we investigated whether **1e** might also show similar kinetics compared to the parent molecule. Derivative **1e** displayed strong concentration-dependent MRSA killing similar in profile to the parent compound [30]. The potency of Derivative **1e** was especially evident at 20× the MIC (7.8 μM), yielding greater than a 4-log kill of MRSA at 4 h (Figure 3B). These killing kinetics parallel the effects previously seen with the natural product parent compound, (−)-**1** [30]. Importantly, the tested concentration of **1e** (MIC 0.39 μM) was half of the concentration of the parent natural product tested in time-kill analyses (MIC 0.75 μM or 0.375 μg/mL) [30]. Secondly at 3.9 μM (10× MIC), bacterial counts were reduced by nearly two log units at three hours incubation and on average by three log units at six hours. In comparison, at six hours of incubation, the parent natural product only yielded a two log decrease in bacterial counts at concentrations two fold higher (7.5 μM) than that tested for Derivative **1e** (3.9 μM) [30]. Furthermore, the actual tested compound concentration of (−)-**1** was 7.5 μM, two fold higher than that of Derivative **1e** (3.9 μM) (Figure 3). In summary, we have discovered a second novel derivative of a natural product with favorable bactericidal activity against MRSA, even in the presence of human serum. These results provide additional data showing that the marinopyrrole A scaffold is amenable to modifications to increase its antibacterial activity.

## 3. Experimental Section

### 3.1. Synthesis of Compounds 5c–6f

All chemicals and solvents were purchased from commercial suppliers and used without further purification. Preparative flash column chromatography was performed on silica gel 60, 0.040–0.063 mm (EMD Chemicals, Billerica, MA, USA). ^1^H NMR (400 MHz) spectra were recorded on a Varian AS400 with a 60-place automated sample changer (Thermo, Madison, WI, USA). High resolution ESI-MS spectra were recorded on an Agilent ESI-TOF LC-MS 6200 system (Agilent Technologies, Santa Clara, CA, USA). Preparative HPLC was performed on a Gilson HPLC system with UV detectors and a Gilson 215 liquid handler for auto injection and fraction collections (customized by HT Labs, San Diego, CA, USA). Analytical HPLC was performed on an Agilent 1100 series with diode array detectors and auto samplers (Agilent Technologies, Santa Clara, CA, USA). The specifications of HPLC analysis are as follows: flow rate, 1 mL/min; column, Intertsil, 2.5 μm, 4.6 × 150 mm; wavelength, 254 and 280 nm; mobile phase, A: H_2_O with 0.1% HCO_2_H, B: MeOH, gradient of 50%–95% B in 25 min. All tested compounds possessed a purity of not less than 95%.

(2-(6-Fluoro-2-methoxybenzoyl)-1′*H*-1,3′-bipyrrol-2′-yl)(2-methoxyphenyl)methanone (**5c**). Into a solution of 6-fluoro-2-methoxybenzoic acid (116 mg, 0.67 mmol, 1.2 equivalent) in benzene (1.0 mL), SOCl_2_ (1.0 mL) was added at room temperature, and the resulting solution was refluxed for 2 h. The reaction mixture was concentrated under vacuum to generate 6-fluoro-2-methoxybenzoyl chloride **4a**, which was used directly in the next step without purification. A solution of **4a** in CH_2_Cl_2_ (DCM, 2 mL) was added to a slurry of AlCl_3_ (97 mg, 1.3 equivalent) in DCM (2.5 mL) at 0 °C, and then, a solution of 1′*H*-1,3′-bipyrrol-2′-yl(2-methoxyphenyl)methanone (**2**) [1] (150 mg, 0.56 mmol, 1.0 equivalent) in DCM (1.5 mL) was added dropwise. The resulting solution was allowed to warm to room temperature and stirred overnight. A saturated solution of NaHCO_3_ (10 mL) and DCM (10 mL) was then added, and the resulting mixture was stirred for 1 h and then filtered through Celite^®^ (Sigma-Aldrich, St. Louis, MO, USA). The mixture was extracted with DCM (3 × 10 mL). The organic layer was dried over anhydrous Na_2_SO_4_, and purified by flash column chromatography (silica gel, hexanes:DCM:EtOAc 4:4:1) to afford 168 mg of **5c** as a white solid, 71% yield. ^1^H NMR (400 MHz, CDCl_3_) δ 9.79 (*br* s, 1H), 7.31–7.27 (m, 1H), 7.22–7.14 (m, 2H), 7.07 (t, *J* = 4.0 Hz, 1H), 6.75–6.68 (m, 4H), 6.54–6.53 (m, 1H), 6.41–6.40 (m, 1H), 6.34 (t, *J* = 4.0 Hz, 1H), 5.70 (dd, *J* = 4.0, 2.6 Hz, 1H), 3.80 (s, 3H), 3.76 (s, 3H). The same procedure as described above was followed to obtain **5d**–**5f**.

(2-(4-Fluoro-2-methoxybenzoyl)-1′*H*-1,3′-bipyrrol-2′-yl)(2-methoxyphenyl)methanone (**5d**). After flash column chromatography (silica gel, DCM:EtOAc 9:1), 113 mg of **5d** was obtained as a yellowish solid, 48% yield. ^1^H NMR (400 MHz, CDCl_3_) δ 9.71 (*br* s, 1H), 7.22–7.14 (m, 3H), 7.07–7.03 (m, 1H), 6.71–6.60 (m, 5H), 6.32 (dd, *J* = 4.0, 1.7 Hz, 1H), 6.29 (t, *J* = 2.8 Hz, 1H), 5.83 (dd, *J* = 4.0, 2.6 Hz, 1H), 3.77 (s, 3H), 3.70 (s, 3H).

(2-(5-Fluoro-2-methoxybenzoyl)-1′*H*-1,3′-bipyrrol-2′-yl)(2-methoxyphenyl)methanone (**5e**). After flash column chromatography (silica gel, DCM:EtOAc 9:1), 118 mg of **5e** was obtained as an off-white solid, 50% yield. ^1^H NMR (400 MHz, CDCl_3_) δ 9.45 (*br* s, 1H), 7.24–7.19 (m, 2H), 7.08–7.04 (m, 2H), 6.89–6.86 (m, 2H), 6.73–6.68 (m, 3H), 6.35–6.32 (m, 2H), 5.85 (dd, *J* = 4.0, 2.6 Hz, 1H), 3.76 (s, 3H), 3.70 (s, 3H).

(2-(5-Bromo-2-methoxybenzoyl)-1′*H*-1,3′-bipyrrol-2′-yl)(2-methoxyphenyl)methanone (**5f**). After flash column chromatography (silica gel, DCM:EtOAc 9:1), 112 mg of **5f** was obtained as a yellowish solid, 48% yield. ^1^H NMR (400 MHz, CDCl_3_) δ 9.71 (*br* s, 1H), 7.45 (dd, *J* = 8.8, 2.5 Hz, 1H), 7.26–7.21 (m, 1H), 7.20–7.15 (m, 2H), 7.07 (t, *J* = 3.0 Hz, 1H), 6.80 (d, *J* = 8.8 Hz, 1H), 6.73 (dt, *J* = 11.3, 8.4 Hz, 3H), 6.34–6.29 (m, 2H), 5.89 (dd, *J* = 4.0, 2.6 Hz, 1H), 3.75 (s, 3H), 3.69 (s, 3H).

(6-Fluoro-2-methoxyphenyl)(4,4′,5,5′-tetrachloro-2′-(2-methoxybenzoyl)-1′*H*-[1,3′-bipyrrole]-2-yl)methanone (**6c**). To a solution of Compound **5c** (150 mg, 0.36 mmol, 1 equivalent) in DCM (4 mL) at 0 °C, SO_2_Cl_2_ (119 μL, 1.48 mmol, 4.1 equivalent) was added dropwise, and the solution was stirred at 0 °C for 1 h. Saturated aqueous NaHCO_3_ solution (2 mL) was added, and the resulting mixture was extracted with DCM (3 × 4 mL). The combined organic layers were dried with anhydrous MgSO_4_, filtered and concentrated. The residue was purified by flash column chromatography (silica gel, DCM:hexane:EtOAc 1:1:0.1) to afford **6c** (118 mg, 59% yield) as an off-white solid. ^1^H NMR (400 MHz, CDCl_3_) δ 10.48 (*br* s, 1H), 7.34 (td, *J* = 8.4, 6.7 Hz, 1H), 7.25–7.18 (m, 2H), 6.80 (d, *J* = 8.4 Hz, 1H), 6.77–6.72 (m, 2H), 6.67 (t, *J* = 7.5 Hz, 1H), 6.37 (s, 1H), 3.80 (s, 3H), 3.77 (s, 3H). 

(4-Fluoro-2-methoxyphenyl)(4,4′,5,5′-tetrachloro-2′-(2-methoxybenzoyl)-1′*H*-[1,3′-bipyrrole]-2-yl)methanone (**6d**). To a solution of compound **5d** (125 mg, 0.30 mmol, 1 equivalent) in DCM (4 mL) at 0 °C, SO_2_Cl_2_ (99 μL, 1.23 mmol, 4.1 equivalent) was added dropwise, and the solution was stirred at 0 °C for 1 h. Saturated aqueous NaHCO_3_ solution (2 mL) was added, and the resulting mixture was extracted with DCM (3 × 4 mL). The combined organic layers were dried with anhydrous MgSO_4_, filtered and concentrated. The residue was purified by flash column chromatography (silica gel, DCM:hexane:EtOAc 1:1:0.1) to afford **6d** (100 mg, 60% yield) as an off-white solid. ^1^H NMR (400 MHz, CDCl_3_) δ 10.60 (*br* s, 1H), 7.27–7.18 (m, 3H), 6.76 (d, *J* = 8.6 Hz, 1H), 6.67 (ddd, *J* = 8.1, 6.2, 3.8 Hz, 3H), 6.32 (s, 1H), 3.80 (s, 3H), 3.72 (s, 3H).

(5-Fluoro-2-methoxyphenyl)(4,4′,5,5′-tetrachloro-2′-(2-methoxybenzoyl)-1′*H*-[1,3′-bipyrrole]-2-yl)methanone (**6e**). To a solution of Compound **5e** (270 mg, 0.64 mmol, 1 equivalent) in DCM (7 mL) at 0 °C, SO_2_Cl_2_ (210 μL, 2.63 mmol, 4.1 equivalent) was added dropwise, and the solution was stirred at 0 °C for 1 h. Saturated aqueous NaHCO_3_ solution (4 mL) was added, and the resulting mixture was extracted with DCM (3 × 4 mL). The combined organic layers were dried with anhydrous MgSO_4_, filtered and concentrated. The residue was purified by flash column chromatography (silica gel, DCM:hexane:EtOAc 4:4:0.5) to afford **6e** (110 mg, 31% yield) as an off-white solid. ^1^H NMR (400 MHz, CDCl_3_) δ 9.81 (*br* s, 1H), 7.28 (dt, *J* = 2.7, 1.8 Hz, 1H), 7.17 (dd, *J* = 7.5, 1.7 Hz, 1H), 7.11 (ddd, *J* = 9.1, 7.9, 3.1 Hz, 1H), 6.88 (ddd, *J* = 8.0, 6.8, 3.6 Hz, 2H), 6.79 (d, *J* = 8.3 Hz, 1H), 6.71 (td, *J* = 7.5, 0.8 Hz, 1H), 6.34 (s, 1H), 3.78 (s, 3H), 3.74 (s, 3H).

(5-Bromo-2-methoxyphenyl)(4,4′,5,5′-tetrachloro-2′-(2-methoxybenzoyl)-1′*H*-[1,3′-bipyrrole]-2-yl)methanone (**6f**). To a solution of Compound **5f** (110 mg, 0.23 mmol, 1 equivalent) in DCM (3 mL) at 0 °C, SO_2_Cl_2_ (75 μL, 0.92 mmol, 4.1 equivalent) was added dropwise, and the solution was stirred at 0 °C for 1 h. Saturated aqueous NaHCO_3_ solution (2 mL) was added, and the resulting mixture was extracted with DCM (3 × 4 mL). The combined organic layers were dried with anhydrous MgSO_4_, filtered and concentrated. The residue was purified by flash column chromatography (silica gel, DCM:hexane:EtOAc 4:4:0.5) to afford **6f** (30 mg, 21% yield) as an off-white solid. ^1^H NMR (400 MHz, CDCl_3_) δ 10.53 (*br* s, 1H), 7.50 (dd, *J* = 8.8, 2.5 Hz, 1H), 7.32–7.27 (m, 1H), 7.20 (dd, *J* = 8.6, 2.1 Hz, 2H), 6.81 (dd, *J* = 14.3, 8.6 Hz, 2H), 6.74 (td, *J* = 7.5, 0.7 Hz, 1H), 6.33 (s, 1H), 3.77 (s, 3H), 3.72 (s, 3H).

### 3.2. Synthesis of Compounds 1c–1f

(6-Fluoro-2-hydroxyphenyl)(4,4′,5,5′-tetrachloro-2′-(2-hydroxybenzoyl)-1′*H*-1,3′-bipyrrol-2-yl)methanone (**1c**). To a solution of **6c** (118 mg, 0.21 mmol) in anhydrous DCM (2 mL) was slowly added 1.0 M solution of BBr_3_ in DCM (848 μL, 0.84 mmol, 4 equivalent) via a syringe under N_2_ at −78 °C. After being stirred for 0.5 h, the mixture was quenched by the addition of MeOH (0.5 mL) and then H_2_O (3 mL) and extracted with DCM (3 × 10 mL). The combined organic layers were dried over anhydrous MgSO_4_, filtered and concentrated in vacuum. The residue was purified by column chromatography (silica gel, hexanes:EtOAc 10:1) to give **1c** (40 mg, 36% yield) as a yellow solid. ^1^H NMR (400 MHz, CDCl_3_) δ 10.47 (s, 1H), 10.01 (s, 1H), 9.80 (*br* s, 1H), 7.43–7.36 (m, 2H), 7.35–7.29 (m, 1H), 6.93 (d, *J* = 8.3 Hz, 1H), 6.82 (d, *J* = 8.5 Hz, 1H), 6.79 (d, *J* = 5.2 Hz, 1H), 6.68–6.62 (m, 1H), 6.49 (t, *J* = 7.6 Hz, 1H). HRMS (ESI-TOF) [M + H]^+^ calcd. for C_22_H_12_Cl_4_FN_2_O_4_ 526.9530, found 526.9521; HPLC purity, 95.1%.

(4-Fluoro-2-hydroxyphenyl)(4,4′,5,5′-tetrachloro-2′-(2-hydroxybenzoyl)-1′*H*-1,3′-bipyrrol-2-yl)methanone (**1d**). To a solution of **6d** (100 mg, 0.18 mmol) in anhydrous DCM (2 mL) was slowly added a 1.0 M solution of BBr_3_ in DCM (720 μL, 0.72 mmol, 4 equivalent) via a syringe under N_2_ at –78 °C. After being stirred for 0.5 h, the mixture was quenched by the addition of MeOH (0.5 mL) and then H_2_O (4 mL) and extracted with DCM (3 × 10 mL). The combined organic layers were dried over anhydrous MgSO_4_, filtered and concentrated in vacuum. The residue was purified by column chromatography (silica gel, hexanes:EtOAc 10:1) to give **1d** (29 mg, 31% yield) as a yellow solid. ^1^H NMR (400 MHz, CDCl_3_) δ 11.60 (s, 1H), 10.41 (s, 1H), 9.84 (*br* s, 1H), 7.57 (dd, *J* = 8.9, 6.5 Hz, 1H), 7.45 (dd, *J* = 8.0, 1.6 Hz, 1H), 7.39–7.34 (m, 1H), 6.93 (d, *J* = 8.4 Hz, 1H), 6.71 (s, *J* = 10.3, 2.5 Hz, 1H), 6.67 (d, *J* = 1.7 Hz, 1H), 6.65–6.58 (m, 1H), 6.56–6.50 (m, 1H). HRMS (ESI-TOF) [M + H]^+^ calcd. for C_22_H_12_Cl_4_FN_2_O_4_ 526.9530, found 526.9533; HPLC purity, 95.2%.

(5-Fluoro-2-hydroxyphenyl)(4,4′,5,5′-tetrachloro-2′-(2-hydroxybenzoyl)-1′*H*-1,3′-bipyrrol-2-yl)methanone (**1e**). To a solution of **6e** (160 mg, 0.29 mmol) in anhydrous DCM (4 mL) was slowly added a 1.0 M solution of BBr_3_ in DCM (1160 μL, 1.16 mmol, 4 equivalent) via a syringe under N_2_ at –78 °C. After being stirred for 0.5 h, the mixture was quenched by the addition of MeOH (0.5 mL) and then H_2_O (5 mL) and extracted with DCM (3 × 10 mL). The combined organic layers were dried over anhydrous MgSO_4_, filtered and concentrated in vacuum. The residue was purified by column chromatography (silica gel, hexanes:EtOAc 10:1) to give **1e** (110 mg, 72% yield) as a yellow solid.^ 1^H NMR (400 MHz, CDCl_3_) δ 10.91 (s, 1H), 10.36 (s, 1H), 9.71 (*br* s, 1H), 7.41–7.36 (m, 2H), 7.27–7.22 (m, 1H), 7.14 (dd, *J* = 8.8, 3.0 Hz, 1H), 6.99 (dd, *J* = 9.1, 4.5 Hz, 1H), 6.94 (d, *J* = 8.4 Hz, 1H), 6.72 (s, 1H), 6.54 (t, *J* = 7.6 Hz, 1H). HRMS (ESI-TOF) [M + H]^+^ calcd. for C_22_H_12_Cl_4_FN_2_O_4_ 526.9530, found 526.9531; HPLC purity, 98.4%.

(5-Bromo-2-hydroxyphenyl)(4,4′,5,5′-tetrachloro-2′-(2-hydroxybenzoyl)-1′*H*-1,3′-bipyrrol-2-yl)methanone (**1f**). To a solution of **6f** (30 mg, 0.05 mmol) in anhydrous DCM (2 mL) was slowly added a 1.0 M solution of BBr_3_ in DCM (196 μL, 0.196 mmol, 4 equivalent) via a syringe under N_2_ at –78 °C. After being stirred for 0.5 h, the mixture was quenched by addition of MeOH (0.5 mL) and then H_2_O (2 mL) and extracted with DCM (3 × 5 mL). The combined organic layers were dried over anhydrous MgSO_4_, filtered and concentrated in vacuum. The residue was purified by column chromatography (silica gel, hexanes:EtOAc 10:1) to give **1f** (12 mg, 42% yield) as a yellow solid. ^1^H NMR (400 MHz, CDCl_3_) δ ^1^H NMR (400 MHz, CDCl_3_) δ 11.12 (s, 1H), 10.35 (s, 1H), 9.82 (s, 1H), 8.10–8.04 (m, 2H), 7.83 (d, *J* = 8.2 Hz, 1H), 7.67 (t, *J* = 7.7 Hz, 1H), 7.58 (dd, *J* = 8.8, 2.3 Hz, 1H), 7.44 (t, *J* = 7.6 Hz, 1H), 6.92 (d, J = 8.9 Hz, 1H), 6.86 (s, 1H). HRMS (ESI-TOF) [M + H]^+^ calcd. for C_22_H_12_BrCl_4_N_2_O_4_ 586.8729, found 586.8731; HPLC purity, 95.6%.

### 3.3. In Vitro Antibacterial Assays

TCH1516, a USA300 strain of community-associated MRSA, was obtained from the American Type Culture Collection (Manassas, VA, USA) and used for biological assays. MICs were determined by broth microdilution in 96-well tissue-culture treated plates (Falcon Becton Dickson, Franklin Lakes, NJ, USA) according to Clinical and Laboratory Standards Institute guidelines, except that Todd-Hewitt broth (THB) was used in place of Mueller-Hinton broth. Vancomycin (NOVAPLUS Hospira, Inc. Lake Forest, IL, USA) served as a control antibiotic. MIC assays in 20% human serum were assessed by bacterial metabolic activity in resazurin, as described [30].

### 3.4. In Vitro Time-Kill Analysis

The bactericidal activity of Derivative **1e** against the MRSA isolate TCH1516 was assessed by time-kill analysis, as described previously [30,31,32,33]. Briefly, MRSA was grown overnight in Todd-Hewitt broth (THB) at 37 °C with shaking. Following overnight growth, MRSA was inoculated in fresh media for growth to the mid-logarithmic phase. At the start of the time-kill assay, bacteria (starting inoculum ~5 × 10^5^ colony forming units (CFU/mL)) were added to duplicate 5-mL polystyrene round-bottom tubes (Falcon, Bedford, MA, USA) containing 20×, 10× or 1× of the MIC of Derivative **1e** (0.39 μM) or an equivalent amount of dimethyl sulfoxide (DMSO, Sigma-Aldrich, St. Louis, MO, USA) vehicle control (Figure 3, none). These cultures were incubated in a shaking 37 °C incubator for 24 h. To determine the rate of antibiotic killing, small aliquots were removed from tubes at 0, 3, 6 and 24 h and serially diluted for CFU enumeration on Todd-Hewitt agar plates (Hardy Diagnostics, Santa Maria, CA, USA). The limit of detection for the time-kill assay was 1.6 (log_10_ CFU/mL).

## 4. Conclusions

In our continuation of studies of novel non-symmetrical derivatives of the marine natural product, marinopyrrole A, we identified a derivative, designated as **1e**, with favorable bactericidal activity against MRSA (MIC = 0.19–0.78 μM). Furthermore, our time-kill studies indicate potent concentration-dependent killing with **1e** that is at least comparable or slightly better than the parent natural product in parallel studies. One of the main drawbacks of the natural product has been its significant reduction in anti-MRSA activity (a 128 to 256 fold increase in MIC) in the presence of human serum. Importantly, **1e** is clearly less serum-inhibited with only a 32–64 fold increase in MIC in 20% human serum (Figure 3). Future derivatization and SAR optimization will continue to identify more potent analogs with activity in human serum.
